# Very highly efficient reduction of CO_2_ to CH_4_ using metal-free N-doped carbon electrodes†
†Electronic supplementary information (ESI) available. See DOI: 10.1039/c5sc04158a


**DOI:** 10.1039/c5sc04158a

**Published:** 2016-01-15

**Authors:** Xiaofu Sun, Xinchen Kang, Qinggong Zhu, Jun Ma, Guanying Yang, Zhimin Liu, Buxing Han

**Affiliations:** a Beijing National Laboratory for Molecular Sciences , Key Laboratory of Colloid and Interface and Thermodynamics , Institute of Chemistry , Chinese Academy of Sciences , Beijing 100190 , China . Email: hanbx@iccas.ac.cn ; Email: qgzhu@iccas.ac.cn

## Abstract

The electrocatalytic reduction of CO_2_ to energy-rich chemicals is a promising pathway for energy storage and utilization. Herein we report the first work on the electrocatalytic reduction of CO_2_ to CH_4_ using metal-free electrodes. It was found that N-doped carbon (graphene-like) material/carbon paper electrodes were very efficient for the electrochemical reaction when using ionic liquids (ILs) as the electrolytes. The faradaic efficiency of CH_4_ could be as high as 93.5%, which is the highest to date. The current density was about 6 times higher than that of a Cu electrode under similar conditions, which is the well-known effective electrode for the electrocatalytic reduction of CO_2_ to CH_4_. Additionally, a trace amount of water in the IL could improve the current density effectively without reducing CH_4_ selectivity considerably. Our results highlight a new class of low-cost and designable electrocatalysts for synthetic fuel production from CO_2_.

## Abstract 2

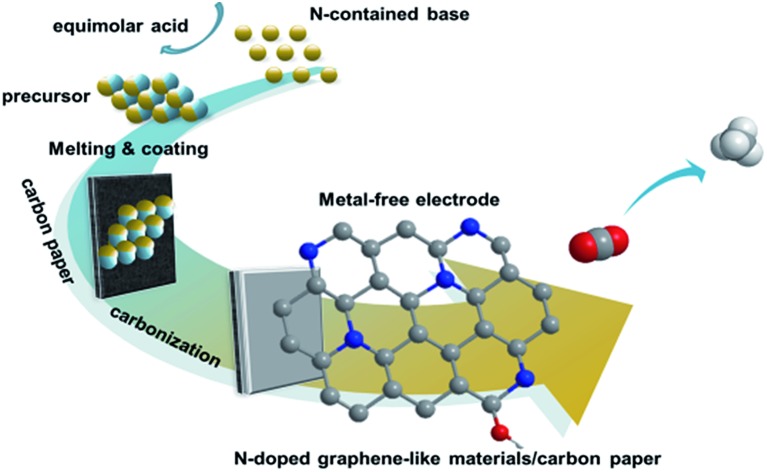
We report the first work on the electrocatalytic reduction of CO_2_ to CH_4_ using metal-free N-doped carbon electrodes.

## Introduction

The conversion of CO_2_ into energy-rich chemicals *via* electrochemical reduction is one of the best ways to use CO_2_ as a carbon resource.^1^–^3^ To date, most studies have used various metals and metal complexes in both aqueous and non-aqueous electrolytes.^4^–^9^ Different chemicals can be produced from CO_2_ electrochemical reduction such as CO, HCOOH, H_2_C_2_O_4_, CH_3_OH, CH_4_, CH_2_CH_2_ and CH_3_CH_2_OH.^10^ However, the study on non-metallic heterogeneous catalysts for CO_2_ electrochemical reduction is very limited. N-doped carbon nanofibers and N-doped carbon nanotubes have been reported for CO_2_ reduction to CO.^11^,^12^ The combination of carbon nanotubes with ammonia plasma treatment and an overlayer of polyethylenimine has been used for the reduction of CO_2_ to formate in aqueous KHCO_3_ solution.^13^ A N-doped nanodiamond/Si rod array electrode has been proposed for the reduction of CO_2_ to acetate with high selectivity.^14^ Non-metal electrodes have some advantages, such as easy design of their composition and structures, excellent chemical stability even in harsh media, and low cost for large-scale practical applications.^15^,^16^ However, the application of this class of electrodes in CO_2_ reduction is at the starting stage, and many interesting phenomena and applications need to be explored.

The electrochemical reduction of CO_2_ to produce CH_4_ is a promising route to produce clean fuel under ambient pressure and temperature, and some elegant works have been conducted by metallic electrodes.^17^–^20^ Compared with the production of many other chemicals, the direct conversion of CO_2_ to CH_4_ with high current density and selectivity is more difficult because of the high C–H bond strength (434 kJ mol^–1^), and an eight electron-transfer step.^10^,^21^

The exploration of efficient routes for the conversion of CO_2_ to CH_4_ is highly desirable and challenging, although extensive work has been conducted. In addition, the use of metal-free electrodes for the electrochemical conversion of CO_2_ to CH_4_ has not been reported.

In recent years, N-doped carbon materials, especially N-doped graphene-like materials (NGMs), and ionic liquids (ILs) have attracted much attention. To satisfy the extensive applications of NGMs in gas capture, catalyst supports, energy storage/conversion, *etc.*, much effort has been focused on the synthetic approaches towards NGMs with controlled structures, regulated textures and heteroatom doping.^22^–^25^ NGMs with different shapes, sizes and chemical compositions have been synthesized *via* the processes of carbonization, hydrothermal carbonization, high-voltage-arc electricity and laser ablation.^26^ ILs, which are molten salts with low melting points, low volatility and high conductivity, have many promising applications.^27^ Especially the imidazolium-based ILs, which exhibit some obvious advantages in the electrochemical reduction of CO_2_ as electrolytes and supporting electrolytes.^1^,^11^

The exploration of efficient non-metal electrolysis systems for CO_2_ reduction is of great importance. Herein we report the first work on the electrochemical reduction of CO_2_ to produce CH_4_ using metal-free electrodes. It was found that NGM electrodes were very efficient for the reaction when using ILs as the electrolytes, and the efficiency depended on the N content in the NGMs significantly. The structure of ILs also affected the electrolysis process, and 1-butyl-3-methylimidazolium tetrafluoroborate ([Bmim]BF_4_) showed excellent performance with a CH_4_ selectivity of 93.5%. Moreover, the addition of a trace amount of water in the IL could increase the current density significantly without reducing the selectivity of CH_4_ considerably.

## Results and discussion

**Fig. 1:**
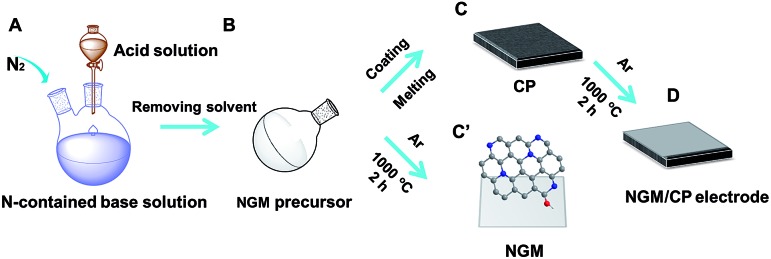
Schematic illustration of the procedures to prepare the NGMs and NGM/CP electrodes.

The procedures to synthesize the NGM/carbon paper (CP) electrodes are shown schematically in Fig. 1, and are similar to those used for the synthesis of NGMs reported in the literature.^25^ The detailed description is provided in the ESI.† Briefly, the N-containing base was mixed with equimolar sulfuric acid in solution (Fig. 1A). The NGM precursor was obtained after removing the solvent and drying (Fig. 1B). The resulting NGM precursor was adhered to one side of the CP (Fig. 1C). The NGM/CP electrode was obtained after carbonation at 1000 °C under an Ar atmosphere (Fig. 1D). The procedure to synthesize the NGMs without the CP was similar, and the only difference was that the NGM precursor was carbonated directly without CP (Fig. 1C′).

The NGMs prepared using 3-pyridinecarbonitrile, 3-hydroxypyridine, 4-dimethylaminopyridine, benzimidazole and 1-vinylimidazole (the structures of these bases are given in ESI Table S1†), are denoted as NGM-1, NGM-2, NGM-3, NGM-4 and NGM-5, respectively (Table 1). The NGMs were characterized by X-ray photoelectron spectroscopy (XPS), elemental analysis, Raman spectroscopy, high-resolution transmission electron microscopy (HR-TEM), X-ray diffraction (XRD), differential scanning calorimetry (DSC), thermal gravimetric analysis (TGA), and N_2_ adsorption/desorption. Some of the results are presented in Table 1. The detailed results and the related discussions are given in the ESI (Fig. S1–S5 and Table S2–S4†). The characterization data showed that the materials were typical NGMs.

**Table 1:** Characteristics of the different NGMs with the total current densities (*j*_tot_) and faradaic efficiencies (FE) for each product over the different NGM/CP electrodes at an applied potential of –1.400 V in bulk [Bmim]BF_4_ with an electrolysis time of 5 h. N_T_ stands for the total N content. N_S_ is the surface N content detected by XPS. *S*_BET_ stands for the specific surface area

Entry	Electrodes	N-Containing base	N_T_/%	N_S_/%	*S*_BET_/m^2^ g^–1^	*j*_tot_/mA cm^–2^	FE_CH_4__/%	FE_CO_/%	FE_H_2__/%
1	CP	—	—	—	—	0.65	0	29.4 ± 0.4	70.5 ± 0.7
2	Graphene/CP	—	0	0	—	0.87	0	32.4 ± 1.9	67.5 ± 1.4
3	NGM-1/CP	3-Pyridinecarbonitrile	9.46	6.52	1.28	1.42	93.5 ± 1.2	4.2 ± 0.2	2.1 ± 0.5
4	NGM-2/CP	3-Hydroxypyridine	6.07	4.34	1.24	1.36	81.6 ± 1.5	5.7 ± 0.6	12.5 ± 0.7
5	NGM-3/CP	4-Dimethylaminopyridine	4.71	3.74	1.09	1.32	67.2 ± 0.4	8.6 ± 1.3	24.6 ± 0.2
6	NGM-4/CP	Benzimidazole	4.03	3.34	0.88	1.27	49.3 ± 0.2	24.5 ± 0.4	26.1 ± 1.5
7	NGM-5/CP	1-Vinylimidazole	3.63	3.17	0.64	1.26	20.8 ± 1.6	56.2 ± 0.3	23.1 ± 0.8

**Fig. 2:**
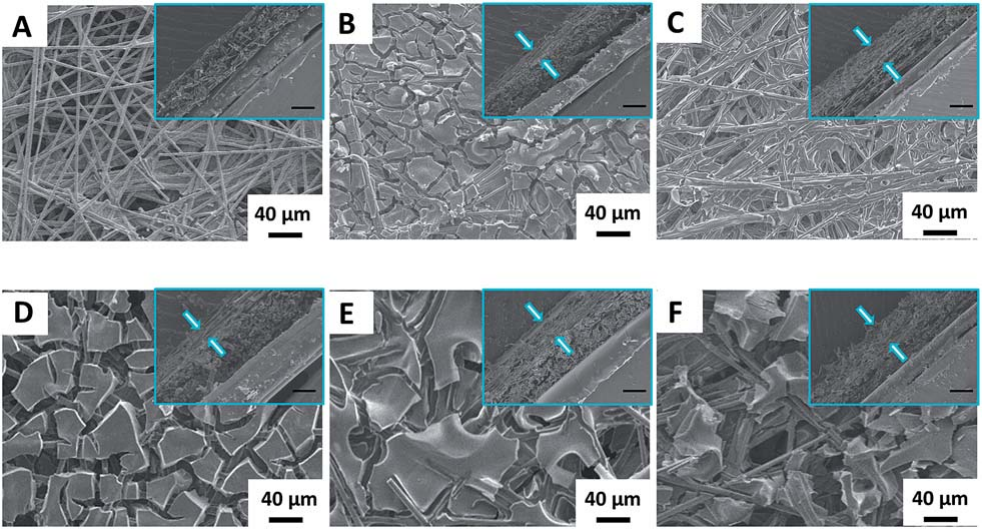
The SEM images of the NGM/CP electrodes. The inset in each image shows the thickness of the NGM on the NGM/CP electrode (scale bar, 30 μm). (A) CP (heated at 1000 °C in an argon (Ar) atmosphere for 2 h), (B) NGM-1/CP, (C) NGM-2/CP, (D) NGM-3/CP, (E) NGM-4/CP and (F) NGM-5/CP.

The morphologies of the NGM/CP electrodes were examined by scanning electron microscopy (SEM). As shown in Fig. 2, the electrodes are coated by an array of striated sheets, which have smooth and nubby surfaces with a thickness of about 30 μm.

The linear sweep voltammetry (LSV) curves (Fig. S6†) show that the NGM/CP electrodes have excellent electrochemical activity towards CO_2_ reduction using bulk [Bmim]BF_4_ as the electrolyte. Controlled potential electrolysis of CO_2_ was then performed in a typical H-cell (Fig. S7†) using NGMs/CP as electrodes and [Bmim]BF_4_ as the electrolyte. The gaseous products contained CH_4_, H_2_ and CO as detected by gas chromatography (GC) and no liquid products were formed as determined by nuclear magnetic resonance (NMR). The total current densities and faradaic efficiencies of CH_4_, CO and H_2_ at –1.400 V are also listed in Table 1. In this work, the potential was measured *versus* the standard hydrogen electrode (SHE). The NGM-1/CP electrode showed the highest CH_4_ faradaic efficiency, which reached 93.5% (entry 3, Table 1). It has been reported that copper is very efficient for CO_2_ reduction to CH_4_.^20^ We also conducted the reaction using a copper electrode and the same electrolyte at different potentials, and the results are compared with that over the NGM-1/CP electrode in Fig. S8 and S9.† Obviously, the faradaic efficiency of NGM-1/CP was much higher than that of the Cu electrode. NGM-1/CP also exhibits about 6 times higher current density compared with the Cu electrode under similar experimental conditions.

Table 1 provides the total N content (N_T_) determined by elemental analysis, and the surface N content (N_S_) obtained from XPS. Both N_T_ and N_S_ decrease from NGM-1 to NGM-5 with the same order. It can be seen that the faradaic efficiency is closely related to the N content of the NGMs (entries 2–7, Table 1). In general, the faradaic efficiency of CH_4_ increased with N_T_ or N_S_. H_2_ was the main product for the graphene/CP electrode, and the amount of CH_4_ formed was negligible as the electrode material had no N. To the best of our knowledge, the unique N content dependence of the faradaic efficiencies and current densities toward CO_2_ reduction has not been reported previously.

We also studied the electrolysis performance using the NGM-1/CP electrode in different ILs and the results are provided in ESI Table S5.† It is interesting to note that the ILs containing fluorine exhibited much higher *j*_tot_ values than those without fluorine, which is partially because fluorine has strong interactions with CO_2_.^28^,^29^ Among the ILs used [Bmim]BF_4_ showed the best performance.

**Fig. 3:**
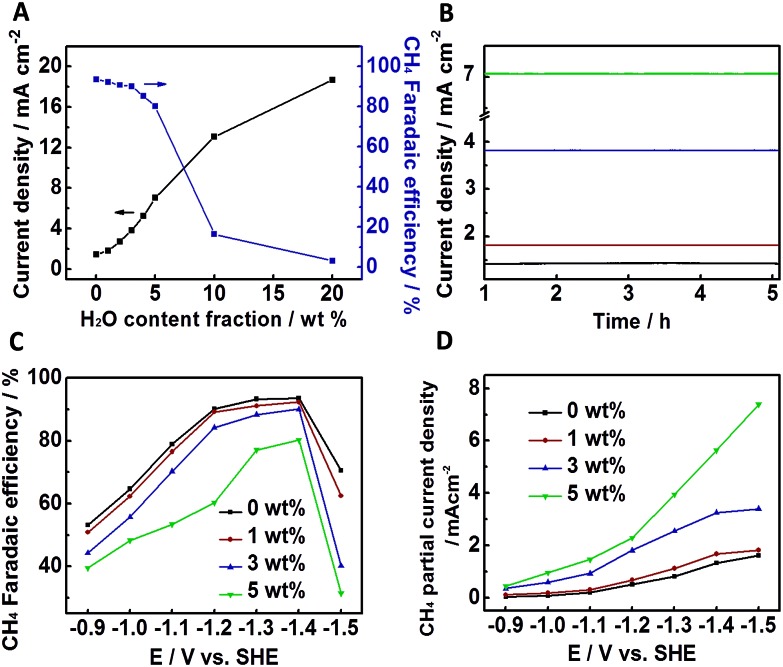
The catalytic performance of the NGM-1/CP electrode for CO_2_ electrochemical reduction using [Bmim]BF_4_–H_2_O binary electrolytes. (A) Current density and CH_4_ faradaic efficiency at an applied potential of –1.400 V. (B) Time curves of the electrolysis processes at water contents of 0 wt%, 1 wt%, 3 wt%, and 5 wt% (from bottom to top) at –1.400 V. (C) Dependence of the faradaic efficiencies of CH_4_ on the applied potential at different water contents. (D) Dependence of the partial current densities of CH_4_ on applied potential at different water contents.

It has been reported that adding water in ILs could improve the electrochemical process effectively.^11^ The results above show that the combination of the NGM-1/CP electrode and [Bmim]BF_4_ is effective for the reduction of CO_2_ to CH_4_. In this work, we also studied the effect of water on the electrolysis, and the results are shown in Fig. 3. Fig. 3A demonstrates that water affected the electrolysis significantly. The current density increased continuously with water content in the IL, but the selectivity of CH_4_ decreased. However, CH_4_ selectivity was still very high when the water content in the IL was less than 5 wt%. In other words, the addition of a trace amount of water could improve the efficiency of the electrolysis effectively. Fig. 3B illustrates the time courses of the electrolysis processes at water contents of 0 wt%, 1 wt%, 3 wt%, and 5 wt%. Obviously, the electrode/electrolyte system had excellent stability. Fig. 3C shows the dependence of the CH_4_ faradaic efficiency on the applied potential at water contents of 0 wt%, 1 wt%, 3 wt%, and 5 wt%. The maximum CH_4_ selectivity occurred at about 1.400 V in each curve. Fig. 3D illustrates the variation of the CH_4_ partial current density, which increased continuously with the potential. Fig. 3C and D indicate that 1.400 V is the most suitable potential for producing CH_4_ because the selectivity towards CH_4_ decreased significantly at higher potential. At a potential of less than 1.400 V, the main by-product was CO, and H_2_ was the main by-product at higher potential (Fig. S10†).

The above results demonstrate that adding a trace amount of water in [Bmim]BF_4_ could enhance the CH_4_ partial current density effectively without decreasing CH_4_ selectivity considerably. Compared with bulk [Bmim]BF_4_, the notable advanced current density in the [Bmim]BF_4_–H_2_O binary electrolytes can be attributed to the decrease in both the pH value and the viscosity of the IL with the addition of water. The former is caused by the formation of [BF_3_OH]^–^, [BF_2_(OH)_2_]^–^ or [BF(OH)_3_]^–^.^11^ At 1.400 V, the CH_4_ partial current density in [Bmim]BF_4_ with 3 wt% water was 3.26 mA cm^–2^, which was about 2.5 times of that in bulk [Bmim]BF_4_, while the selectivity of CH_4_ was still as high as 90.1%.

The mechanism of the electrochemical reduction of CO_2_ to CH_4_ on metal electrodes has been discussed. An adduct of CO_2_–CO_2_˙^–^ may be formed over metals, especially in non-aqueous solvents.^30^ A density functional theory (DFT) study suggested that the key potential determining step in the formation of CH_4_ is the hydrogenation of adsorbed CO to form CHO_ads_.^17^,^31^ It is very likely that CHO_ads_ is the key intermediate towards the breaking of the C–O bond, leading to the formation of CH_4_.^6^

In the NGMs of this work, the N atoms are polarized negatively due to electron-withdrawing effects in the graphene π system, with positively charged C atoms.^32^–^34^ The XPS spectra of the NGMs (Fig. S2†) show that there exists pyridinic, pyridonic/pyrrolic, and quaternary N species in the carbon lattice (Fig. S3†). The pyridinic and pyridonic/pyrrolic N species are dominant, and have previously been shown to act as electrochemically active species in the reduction of CO_2_ to CO and CH_3_OH.^11^,^35^,^36^ It can be seen from Table 1 and S4† that the catalytic activity of the NGMs decreased with decreasing contents of these active N species, mainly because the N sites can interact strongly with CO_2_ and the intermediates in the reaction.

As discussed above, the pyridinic and pyridonic/pyrrolic N species are the main active species for the electrochemical reaction. In order to show the effect of the active N species on the selectivity clearly, Table S6† gives the active N species (calculated from Table 1 and S4†) and the product selectivity. As the content of the active N species changes from 1.8% to 4.8%, the faradaic efficiency of CH_4_ increases from 20.8% to 93.5%, while the faradaic efficiency of CO decreases from 56.2% to 4.2%. This indicates that the active N species play a crucial role for the high selectivity of CH_4_. It has been reported that other products, such as CO, were obtained over N-doped carbon nanofibers and N-doped carbon nanotubes in other electrolytes.^11^,^12^ One of the main reasons is that the IL in the electrolyte also plays a very important role for producing CH_4_ (Fig. 3A), and this work makes the first combination of NGMs and ILs to produce CH_4_ and very high selectivity can be achieved.

**Fig. 4:**
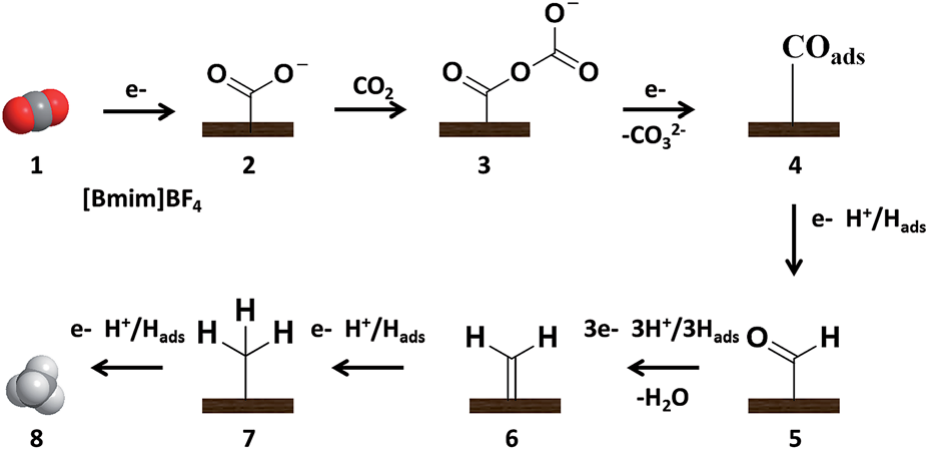
CO_2_ reduction mechanism schematic diagram at NGM/CP electrode.

On the basis of the experimental results of this work and the related knowledge discussed above, we propose a possible mechanism for the electrochemical reduction of CO_2_ to CH_4_ on the NGM electrodes, which is shown schematically in Fig. 4. The CO_2_ is first adsorbed to the pyridinic N and pyridonic/pyrrolic N binding sites, where it is reduced to CO_2_˙^–^ (**2**). In this step, [Bmim]BF_4_ helps to drive the transformation of CO_2_ to **2**. Then, CO_2_˙^–^ is coupled with a Lewis acidic CO_2_ molecule from solution to form CO_2_–CO_2_˙^–^ (**3**).^30^ The free-energy pathway becomes thermodynamically downhill to transfer the second electron to form adsorbed CO_ads_ (**4**). The CO_ads_ can be desorbed or converted into CHO_ads_ after accepting an electron and proton (**5**). The CHO_ads_ can be transformed to CH_4_ after accepting additional electrons and protons (**6–8**), which is similar to that on Cu electrodes.^31^ It is known that CO_ads_ interacts more strongly with the N sites than the C sites in NGMs.^12^,^13^,^37^,^38^ The strong interaction between CO_ads_ and the electrode can prevent the escape of CO from the electrode, which is favorable for its further hydrogenation to form CH_4_.

## Conclusions

In summary, a series of NGMs with different N contents have been prepared on CP and used as electrodes for CO_2_ electrochemical reduction. The metal-free NGM/CP electrodes exhibit excellent activity and selectivity for the electrochemical transformation of CO_2_ to CH_4_ using ILs as the electrolytes. The faradaic efficiency of CH_4_ increased significantly with increasing N content in the NGMs. The faradaic efficiency of CH_4_ can be as high as 93.5% in a NGM-1/CP electrode–[Bmim]BF_4_ system. The current density can increase from 1.42 to 3.26 mA cm^–2^ when 3 wt% water is added in the IL, and the faradaic efficiency of CH_4_ is still as high as 90.1%. The NGM-1/CP electrode shows about 6 times higher current density compared with the Cu electrode under similar experimental conditions. We believe that by taking the advantages of non-metal materials, such as their easily designable composition and structures, excellent chemical stability, and low cost, many novel electrodes can be designed for CO_2_ electrochemical conversion.

## Supplementary Material

Supplementary information
