# Supplementary material for: Very highly efficient reduction of CO2 to CH4 using metal-free N-doped carbon electrodes
Source: Chem Sci. 2016 Jan 15;7(4):2883–7. doi: 10.1039/c5sc04158a (PMC6054036; doi:10.1039/c5sc04158a)
Supplement: Supplementary file 1 [file SC-007-C5SC04158A-s001.pdf]

Electronic Supplementary Material (ESI) for Chemical Science.
This journal is © The Royal Society of Chemistry 2016

Electronic Supplementary Information

Very highly efficient reduction of CO_2 to CH_4 using metal-free N-doped carbon electrodes

Xiaofu Sun, Xinchen Kang, Qinggong Zhu*, Jun Ma, Guanying Yang, Zhimin Liu, Buxing Han*

Beijing National Laboratory for Molecular Sciences, Institute of Chemistry, Chinese Academy of Sciences, Beijing 100190, China

1. Experimental Section

Materials

3-Pyridinecarbonitrile (purity>98%) and 1-vinylimidazole (purity>99%) were purchased from Sigma Aldrich. 3-Hydroxypridine (purity>99%), benzimidazole (purity>98%), 4-dimethylaminopyridine (purity>99%) were obtained from TCI. The structures of these bases are given in Table S1. Sulfuric acid （95-98%） and Cu foil (purity>99.99%) were provided by Sinopharm Chem. Reagent Co. Ltd. Ionic liquids, 1-butyl-3-methylimidazolium tetrafluoroborate ([Bmim]BF_4, purity>99%), 1-butyl-3-methylimidazolium hexafluorophosphate ([Bmim]PF_6, purity>99%), 1-butyl-3-methylimidazolium trifluoromethanesulfonate ([Bmim]TfO, purity>99%), 1-butyl-3-methylimidazolium bis(trifluoromethylsulfonyl)imide ([Bmim]Tf_2N, purity>99%) and 1-butyl-3-methylimidazolium dicyanamide ([Bmim]DCA, purity>99%) were purchased from the Centre of Green Chemistry and Catalysis, LICP, CAS. Toray Carbon Paper (CP, TGP-H-60, 19×19 cm), Nafion D-521 dispersion (5 % w/w in water and 1-propanol, ≥ 0.92 meg/g exchange capacity) and Nafion N-117 membrane (0.180 mm thick, ≥ 0.90 meg/g exchange capacity) were purchased from Alfa Aesar China Co., Ltd. The commercial graphene was obtained from Sinocarbon Graphene Marketing Center, Chinese Academy of Sciences.

Synthesis of N-doped graphene-like materials (NGMs) and preparation of NGM/CP electrodes

The procedures were similar to that reported by other authors.[1, 2] In a typical experiment, 50 mmol of the N-containing base was dissolved in 20 mL deionized water (for 3-pyridinecarbonitrile and benzimidazole) or methanol (for 1-vinylimidazole, 4-dimethylaminopyridine and 3-hydroxypridine due to their low solubility in water) in a round-bottom flask. Equimolar sulfuric acid aqueous solution (1.75 mol/L) was gradually added into the flask in an ice bath under continuous stirring and a N_2 atmosphere. Then, mixture was stirred for 3 h at 25 °C. The solvent was removed by rotary evaporation at 80 °C under vacuum, and the NGM precursor was obtained. Each of precursors with a certain amount (it was calculated to make sure the amount of the final carbon material was about 1 mg according to the thermogravimetric data) was uniformly adhered to one side the CP (1 cm × 1 cm) on a porcelain boat, which was heated in the tube furnace at 10 °C min^{-1} in argon (Ar) atmosphere to 1000 °C and kept at this temperature for 2 h. The procedures to obtain the NGMs without carbon paper were similar. The main difference was that the NGM precursor was carbonated directly without CP.

The method to prepare the Graphene/CP electrode was similar to that reported,[2,3] which is described briefly as follows. 1 mg commercial graphene was suspended in 1 mL ethanol with 10 μL Nafion D-521 dispersion (5 wt%) to form a homogeneous ink assisted by ultrasound. Then, it was spread onto the CP (1 cm × 1 cm) surface by a micropipette and dried under room temperature. Here the CP was heated at 1000 °C in argon (Ar) atmosphere in 2 h.

Materials characterization

^{1}H and ^{13}C NMR spectra of the precursors of NGMs were recorded on a Bruker Avance III 400 HD spectrometer operating at 400 MHz for ^{1}H and 100 MHz for ^{13}C in d_6-DMSO with TMS as an internal standard.

X-ray photoelectron spectroscopy (XPS) analysis was performed on the Thermo Scientific ESCALab 250Xi using 200 W monochromatic Al Kα radiation. The 500 μm X-ray spot was used for XPS analysis. The base pressure in the analysis chamber was about 3×10^{-10} mbar. Typically, the hydrocarbon C1s line at 284.8 eV from adventitious carbon is used for energy referencing.

The Raman spectra of the NGMs were obtained at room temperature in flame-sealed capillary on a FT Bruker RFS 106/S spectrometer, equipped with a 514 nm laser, in the region from 4000 to 100 cm^{-1} with a resolution of 2 cm^{-1}.

Powder X-ray diffraction (XRD) patterns were collected on the X-ray diffractometer (Model D/MAX2500, Rigaka) with Cu-Kα radiation.

Thermogravimetric (TG) measurements were conducted using a Pyris 1 thermo-gravimetry analyzer from room temperature to 700 °C at a heating rate of 10 °C·min^{-1} under a N_2 flow of 60 mL·min^{-1} with open alumina pans.

Differential scanning calorimetry (DSC) (Q-2000 TA Instruments) was used to determine the melting points of the NGMs at a heating rate of 10 °C/min under N_2 atmosphere.

The N_2 adsorption/desorption isotherms of the NGMs were determined using a Quadrasorb SI-MP system.

The morphologies of NGM/CP electrodes were characterized by a HITACHI S-4800 scanning electron microscope (SEM) and a JEOL JEM-2100F high-resolution transmission electron microscopy (HR-TEM).

linear sweep voltammetry (LSV) study

The electrochemical workstation (CHI 6081E, Shanghai CH Instruments Co., China) was used. LSV measurements were carried out in a single compartment cell with three-electrode configuration, which consisted of working electrode, a platinum gauze auxiliary electrode, and an Ag/Ag^+ (0.01 M $AgNO_3$ in 0.1 M TBAP-MeCN) reference electrode for IL or IL-based electrolytes. Before each experiment, all the

electrodes were sonicated in acetone for 10 min and then washed with water and ethanol, followed by drying in N_2 atmosphere. In a typical experiment, the electrolyte was bubbled with N_2 or CO_2 for at least 30 min to form N_2 or CO_2 saturated solution. LSV measurement in gas-saturated electrolyte was conducted in the potential range of 0.6 to -1.6 V versus the standard hydrogen electrode (SHE) at a sweep rate of 20 mV/s. Slight magnetic stirring was applied in the process.

CO_2 reduction electrolysis and product analysis

The electrolysis experiments were conducted at 25 °C in a typical H-type cell with an Ag/Ag^+ reference electrode. The apparatus was similar to that used by other researchers,[3] and is schematically shown in Figure S6. In the experiments, the cathode and anode compartments were separated through a Nafion 117 proton exchange membrane. The IL (or IL-water) and H_2SO_4 aqueous solution (0.5 M) were used as cathodic and anodic electrolytes, respectively. Under the continuous stirring, CO_2 was bubbled through the catholyte (2 mL/min) for 30 min before electrolysis. Then, potentiostatic electrochemical reduction of CO_2 was carried out with CO_2 bubbling (2 mL/min), and the gaseous product was collected using a gas bag and analyzed by gas chromatography (GC, HP 4890D), which was equipped with TCD and FID detectors using helium as the internal standard. The liquid product was analyzed in DMSO-d_6 with TMS as an internal standard by ^1H NMR (Bruker Avance III 400 HD spectrometer). The Faradaic efficiency of the products were calculated from GC analysis data.[4] The experiments were run at different potentials.

2. Results and discussion

TGA and DSC study: Fig. S1 shows the thermal gravimetric analysis curves of the NGM procedures. The data indicated that the NGM procedures had different thermal stability. However, all of them were stable below 200 ^{o}C. Table S2 shows the melting points of the NGM procedures determined by DSC, which show that the NGM procedures were solid at the experimental temperature of the electrochemical investigations.

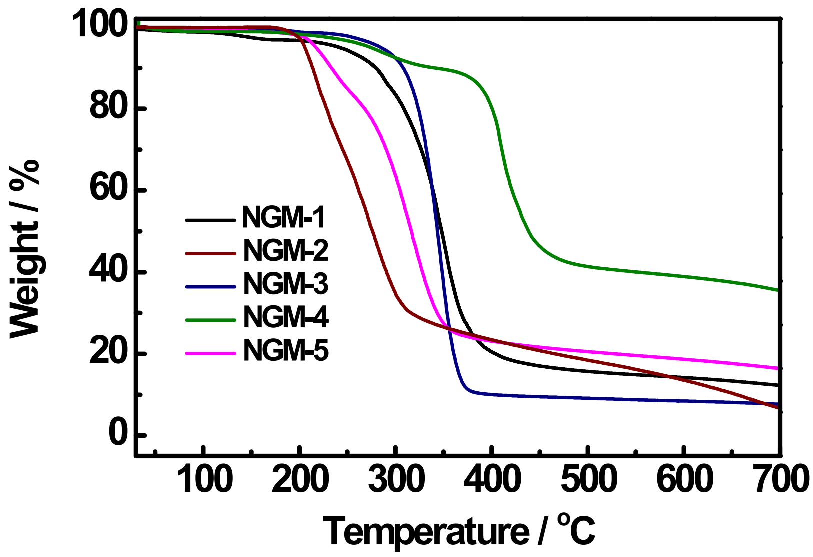

Fig. S1. Thermal gravimetric analysis curves of the NGM procedures.

XPS study: The XPS spectra of NGMs are shown in Fig. S2. It can be known that N atoms are integrated into the structure of the materials. Taking NGM-1 as an example, the N1s peak can be resolved into three compositions at binding energies of 398.0 eV (pyridinic N), 400.0 eV (pyridonic and pyrrolic N) and 401.3 eV (quaternary N), respectively (inset in Figure S2A).[5] The extent of the doped N can be controlled by varying precursors.

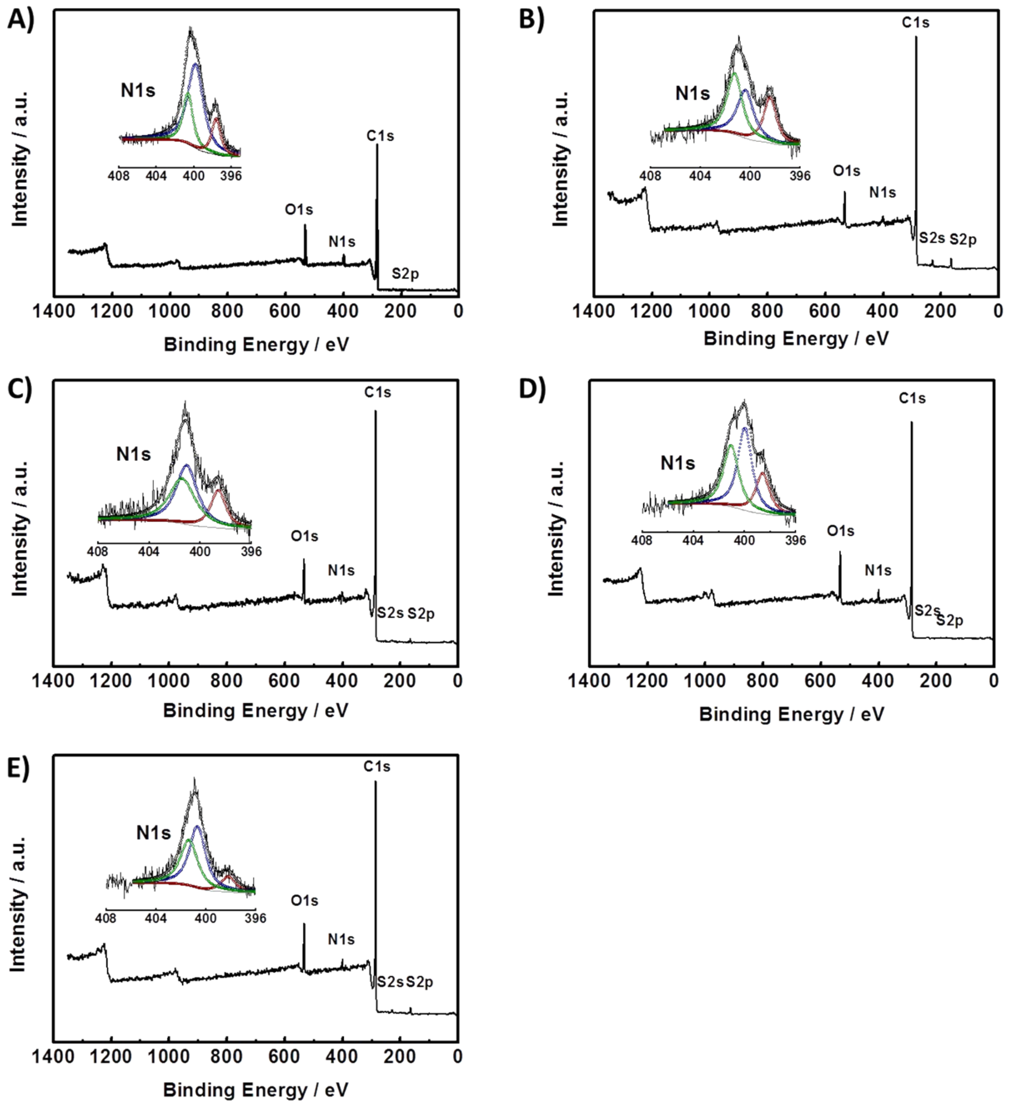

Fig. S2. Fitting of N1s core level XPS spectra of different NGMs, including NGM-1 (A), NGM-2 (B), NGM-3 (C), NGM-4 (D) and NGM-5 (E).

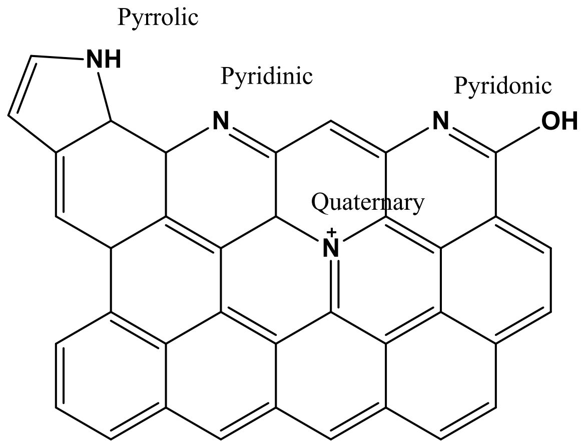

Fig. S3. Structural models of N species in various chemical environments in NGMs.

Raman spectroscopy study: In Raman spectra of NGMs, D band is a breathing mode with A1g symmetry involving phonons near the K zone boundary. In the perfect graphite structure, it is forbidden. But D band becomes active in the presence of defects such as edges, structural disorders and functional groups.[6] It has been reported that the distortion in the carbon hexagonal lattice may lead to the increase in the intensity of D band. In the meantime, G band respects E2g symmetry that involves the in-plane bond-stretching motions of pairs of sp^2 C, which does not require the presence of hexatomic ring.[2] In fact, the band position, the intensity ratio of D band and G band (I_D/I_G), as well as the full width at half maximum (FWHM) of D band or G band are reported to be related to the disorder or defects in the graphitic structure.[6]

Fig. S4 shows the Raman spectra of the NGMs. It can be seen that the positions of D band and G band and I_D/I_G are similar for the NGMs. The ratios of I_D/I_G in the Raman spectra of NGMs were in the range of 0.97-1.05, indicating the presence of defects in the typical graphite carbon lattice.

The TEM image of the typical NGM is shown in inset of Fig. S4. It can be known that the NGM is made up of bent graphite-like sheets, which is similar to those of the N-doped NGMs reported by other authors.[2].

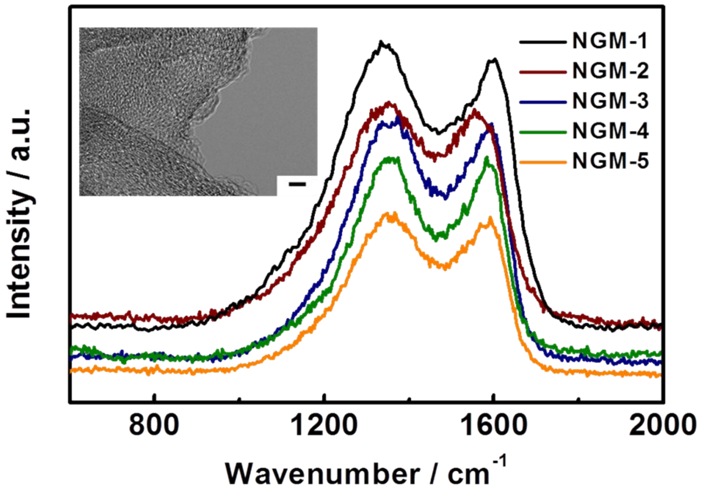

Fig. S4. Raman spectra of the NGMs. Inset: HR-TEM image of NGM-1 (scale bar, 2 nm).

XRD study: Fig. S5 gives the XRD patterns of different NGMs. Judging from the Bragg reflection of the (002) peak, the present NGMs with high N content display a more positive shift of the diffraction position. In particular, NGM-1 shows a high-intensity (002) diffraction peak centered at 26.2°, along with clearly observable (100) and (110) reflection characteristics for graphitic structures. The calculated interlayer distance (d_{002}) of 0.343 nm is very close to that of graphite.[7] The values of d_{002} of NGM-2, NGM-3, NGM-4 and NGM-5 are 0.348, 0.367, 0.373 and 0.366 nm, respectively. Compared with the common GMs, the doping effect of electron-rich N can induce a donor state above the Fermi energy level and increase its electron density, especially the NGMs with high degree of graphitization and high N content.[2]

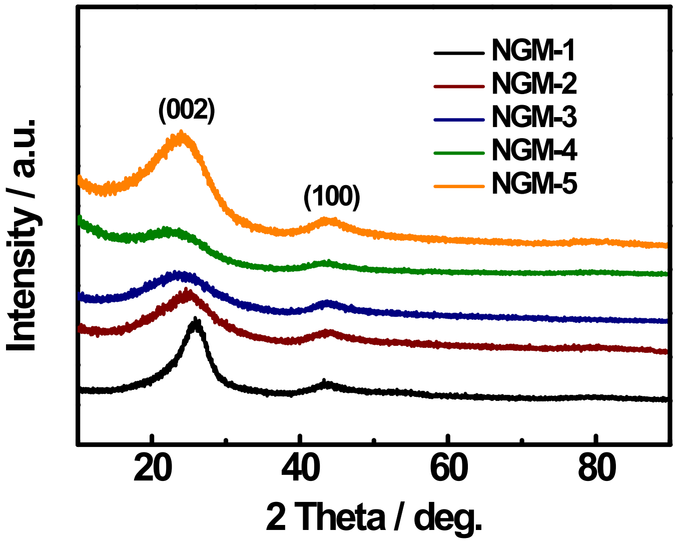

Fig. S5. XRD patterns of different NGMs.

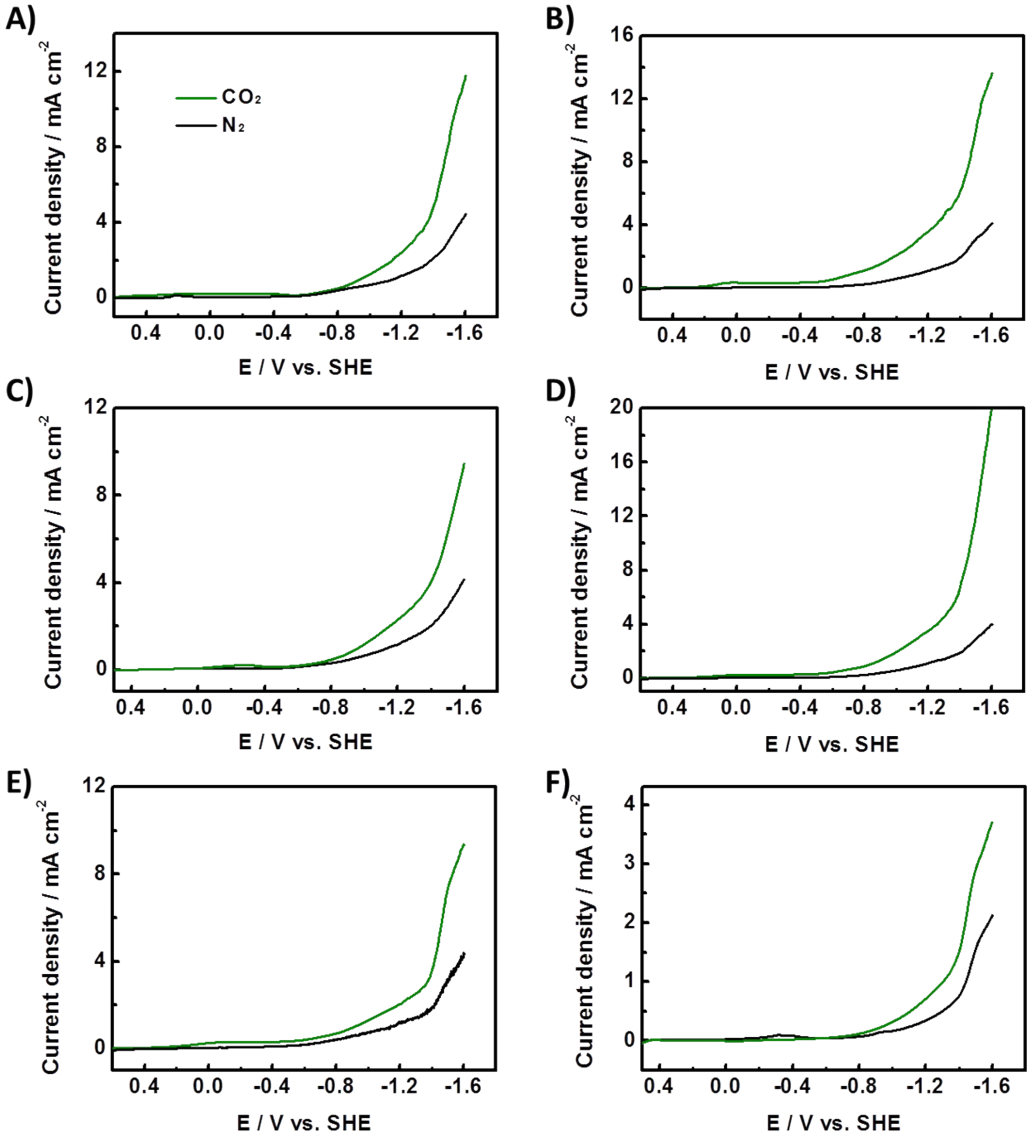

Fig. S6. LSV curves in pure [Bmim]BF_4 over different NGM/CP electrodes, including NGM-1/CP (A), NGM-2/CP (B), NGM-3/CP (C), NGM-4/CP (D), NGM-5/CP (E) and CP (F).

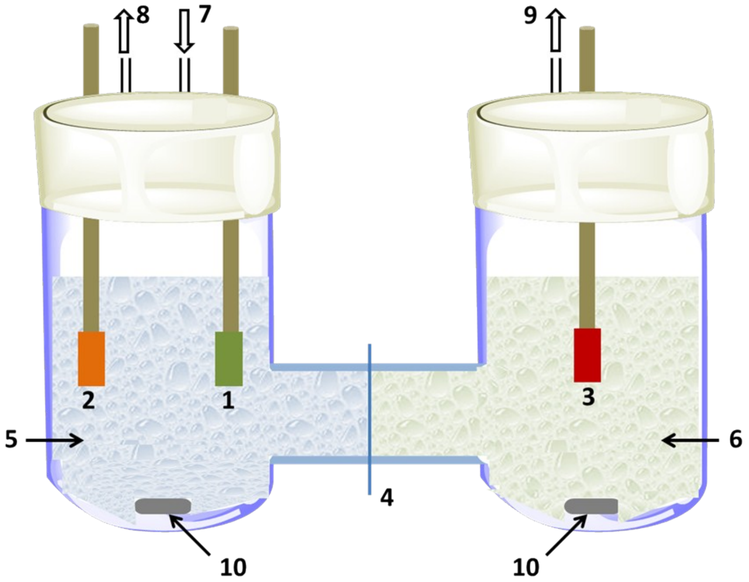

Fig. S7. The schematic diagram of the electrolysis device. 1. the working electrode, 2. the reference electrode, 3. the auxiliary electrode, 4. Nafion 117 membrane, 5. the electrolyte, 6. 0.5 mol/L H_2SO_4 aqueous solution, 7. CO_2 inlet, 8. the gas product outlet, 9. O_2 outlet, 10. magnetic stirrer.

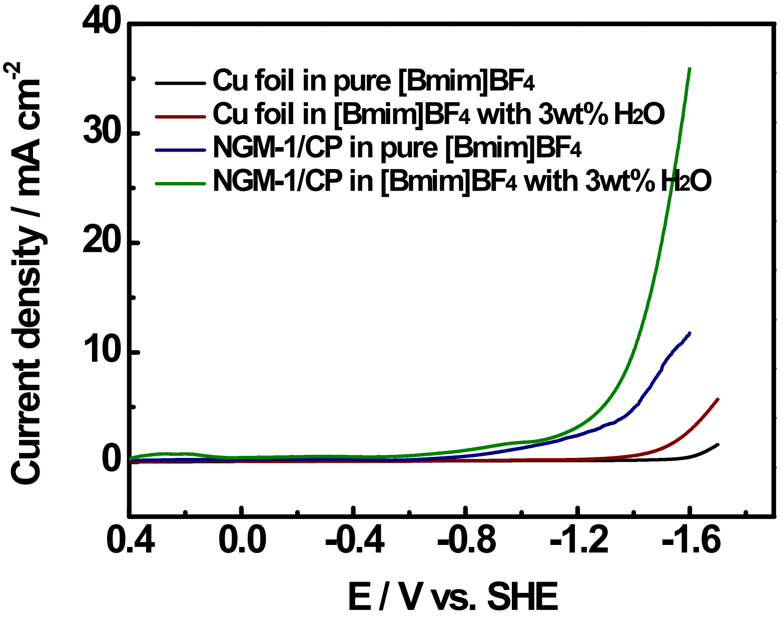

Fig. S8. LSV curves of Cu foil and NGM-1/CP electrodes in CO_2-saturated pure $[Bmim]BF_4$ and CO_2-saturated $[Bmim]BF_4$ with 3 wt % H_2O.

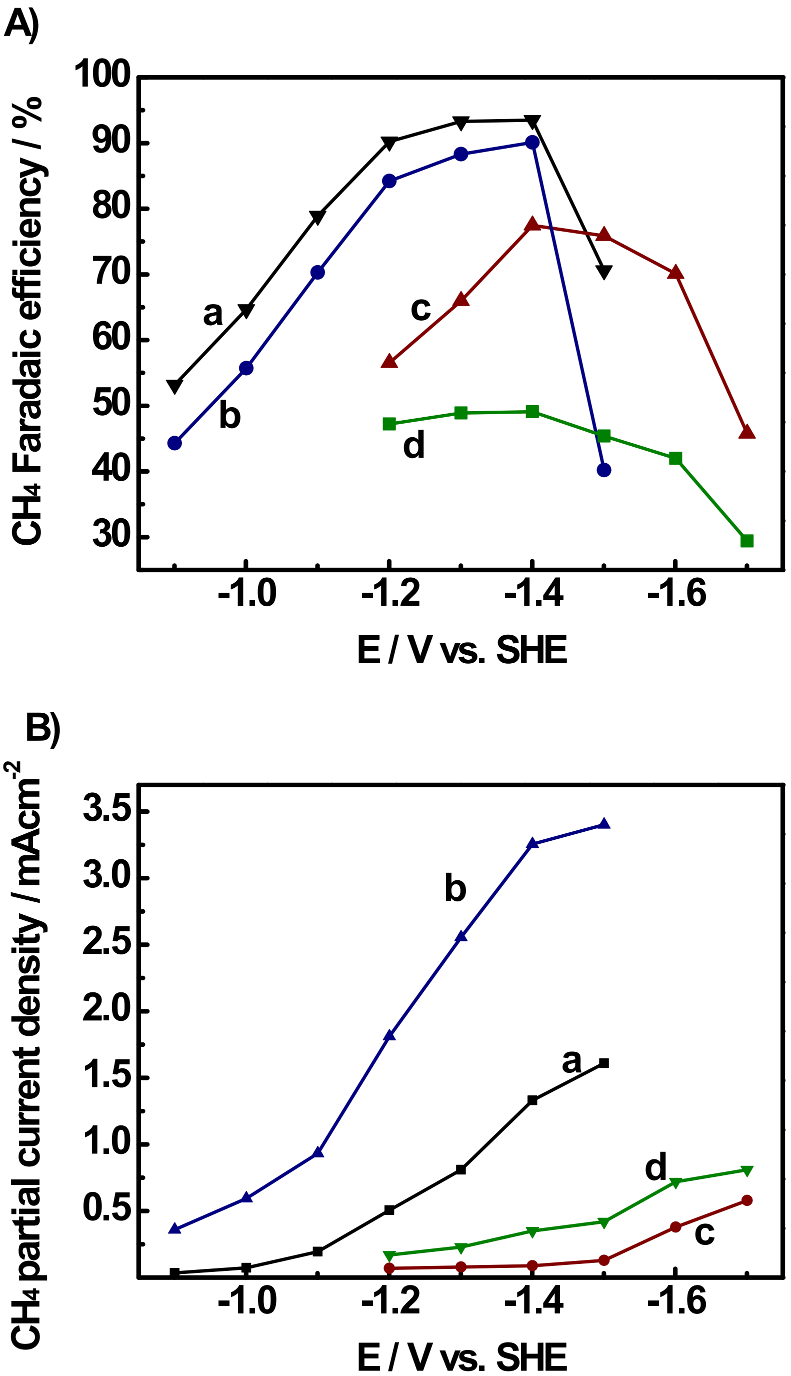

Fig. S9. A) Dependence of Faradaic efficiencies of CH_4 on applied potential in CO_2-saturated pure [Bmim]BF_4 on NGM-1/CP electrode (a), CO_2-saturated [Bmim]BF_4 with 3 wt % H_2O on NGM-1/CP electrode (b), CO_2-saturated pure [Bmim]BF_4 on Cu foil electrode (c), and CO_2-saturated [Bmim]BF_4 with 3 wt % H_2O on Cu foil electrode (d). B) Dependence of partial current densities of CH_4 on applied potential in CO_2-saturated pure [Bmim]BF_4 on NGM-1/CP electrode (a), CO_2-saturated [Bmim]BF_4 with 3 wt % H_2O on NGM-1/CP electrode (b), CO_2-saturated pure [Bmim]BF_4 on Cu foil electrode (c), and CO_2-saturated [Bmim]BF_4 with 3 wt % H_2O on Cu foil electrode (d).

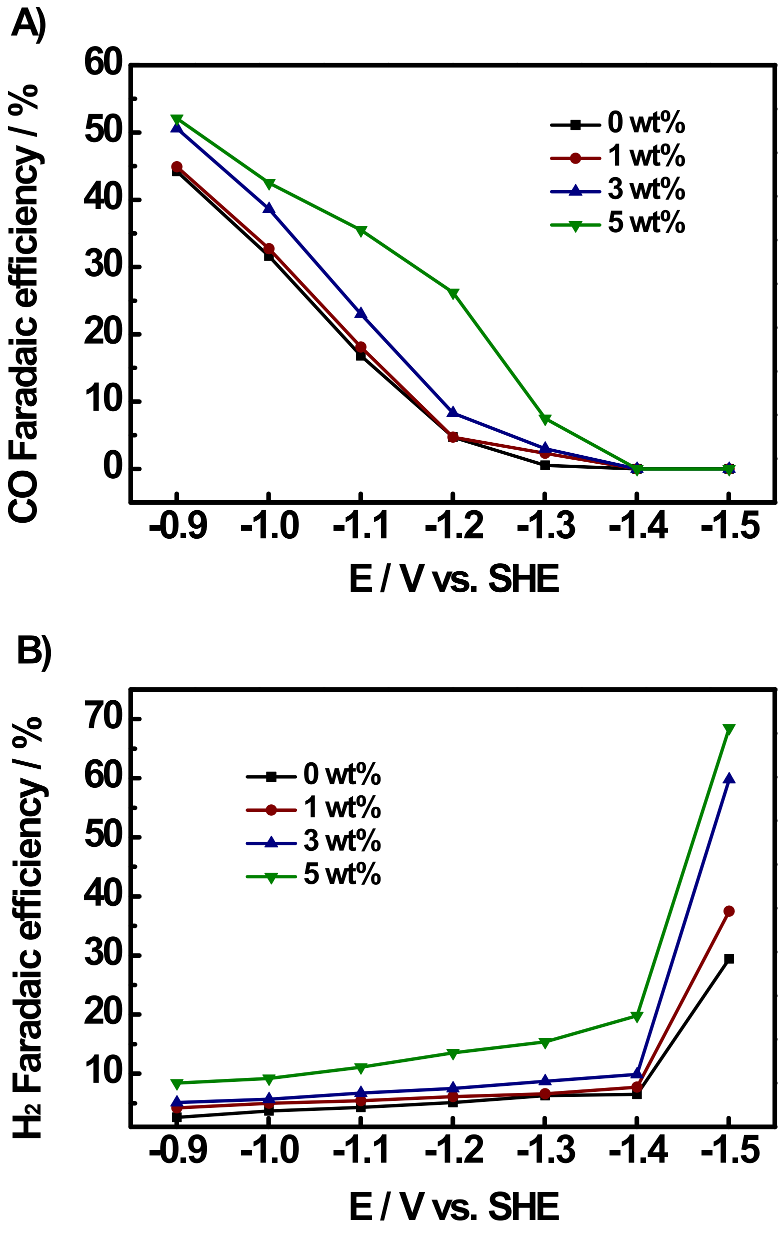

Fig. S10. Faradaic efficiencies for CO (A) and H_2 (B) over NGM-1/CP electrode in [Bmim]BF_4 with different water contents at various applied potentials.

Table S1 Structure of the N-containing bases used in this work.

No.	Name	Structure
1	3-Pyridinecarbonitrile	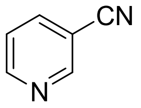
2	3-Hydroxypyridine	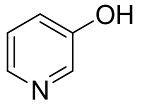
3	4-Dimethylaminopyridine	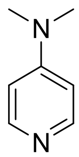
4	Benzimidazole	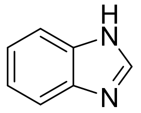
5	1-Vinylimidazole	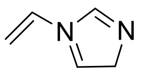

Table S2 The melting points (Tm) of different NGM procedures obtained from DSC study.

Entry	Materials	Tm / °C
1	NGM-1	98.4
2	NGM-2	127.9
3	NGM-3	164.8
4	NGM-4	188.5
5	NGM-5	73.1

Table S3. The element composition of the NGMs detected by elemental analysis.

Materials	C / %	N / %	S / %
NGM-1	84.17	9.46	2.53
NGM-2	86.66	6.07	1.97
NGM-3	86.11	4.71	2.71
NGM-4	86.21	4.03	2.52
NGM-5	87.39	3.63	2.32

Table S4. Relative contents of different N species in the NGMs obtained from the XPS spectra.

Materials	pyridinic N / %	pyridonic and pyrrolic N / %	quaternary N / %
NGM-1	22.2	51.4	26.4
NGM-2	31.7	32.2	36.1
NGM-3	23.5	39.2	37.3
NGM-4	17.3	43.5	39.2
NGM-5	9.5	47.2	43.3

Table S5. The total current densities (j_{tot}) and FE for each product (CH_4, CO, H_2) of NGM-1/CP electrode for the reduction of CO_2 at an applied potential of -1.400 V vs SHE in various kinds of ILs with an electrolysis time after 5 h.

Electrolytes	j_{tot} / mA cm^{-2}	FE_{CH4} / %	FE_{CO} / %	FE_{H2} / %
[Bmim]BF_4	1.424	93.5±1.2	4.2±0.2	2.1±0.5
[Bmim]PF_6	0.372	91.1±0.3	4.0±1.0	3.7±0.7
[Bmim]TfO	0.698	93.1±0.6	1.0±0.4	4.5±0.7
[Bmim]Tf_2N	0.656	96.0±0.2	0±0.5	3.0±0.3
[Bmim]DCA	0.214	93.1±0.3	3.4±1.1	3.0±0.4

Table S6. The contents of active N (pyridinic or pyridonic or pyrrolic N) in the electrode materials and product selectivity.

Active N/ %	Main product and Faradaic efficiency	Refs
Not reported	CO, 98%	8[a]
2.6	CO, 80%	9[b]
4.80	CH_4, 93.5%; CO, 4.2%	This work
2.77	CH_4, 81.6%; CO, 5.7%	This work
2.34	CH_4, 67.2%; CO, 8.6%	This work
2.03	CH_4, 49.3%; CO, 24.5%	This work
1.80	CH_4, 20.8%; CO, 56.2%	This work

a. N-doped carbon nanofiber/ionic liquid system

b. N-doped carbon nanotubes/$KHCO_3$(aq) system.

References

1. S. Zhang, K. Dokko and M. Watanabe, *Chem. Mater.*, 2014, **26**, 2915.
2. S. Zhang, M. S. Miran, A. Ikoma, K. Dokko and M. Watanabe, *J. Am. Chem. Soc.*, 2014, **136**, 1690.
3. D. Gao, H. Zhou, J. Wang, S. Miao, F. Yang, G. Wang, J. Wang and X. Bao, *J. Am. Chem. Soc.*, 2015, **137**, 4288.
4. B. A. Rosen, A. Salehi-Khojin, M. R. Thorson, W. Zhu, D. T. Whipple, P. J. A. Kenis and R. I. Masel, *Science*, 2011, **334**, 643.
5. P. Chen, L. K. Wang, G. Wang, M. R. Gao, J. Ge, W. J. Yuan, Y. H. Shen, A. J. Xie and S. H. Yu, *Energy Environ. Sci.*, 2014, **7**, 4095.
6. L. Qu, Y. Liu, J. B. Baek and L. Dai, *ACS Nano*, 2010, **4**, 1321.
7. Y. Gogotsi, A. Nikitin, H. Ye, W. Zhou, J. E. Fischer, B. Yi, H. C. Foley and M. W. Barsoum, *Nat. Mater.*, 2003, **2**, 591.
8. B. Kumar, M. Asadi, D. Pisasale, S. Sinha-Ray, B. A. Rosen, R. Haasch, J. Abiade, A. L. Yarin and A. Salehi-Khojin, *Nat. Commun.*, 2013, **4**, doi:10.1038/ncomms3819.
9. J. Wu, R. M. Yadav, M. Liu, P. P. Sharma, C. S. Tiwary, L. Ma, X. Zou, X.-D. Zhou, B. I. Yakobson, J. Lou and P. M. Ajayan, *ACS Nano*, 2015, **9**, 5364.
